# Bioassay-Guided Isolation, Metabolic Profiling, and Docking Studies of Hyaluronidase Inhibitors from *Ravenala madagascariensis*

**DOI:** 10.3390/molecules25071714

**Published:** 2020-04-08

**Authors:** Esraa M. Mohamed, Mona H. Hetta, Mostafa E. Rateb, Mohamed A. Selim, Asmaa M. AboulMagd, Farid A. Badria, Usama Ramadan Abdelmohsen, Hani A. Alhadrami, Hossam M. Hassan

**Affiliations:** 1 Department of Pharmacognosy, Faculty of Pharmacy, MUST, Giza 12566, Egypt; esraakadrymohamed@gmail.com (E.M.M.); selim2010@hotmail.com (M.A.S.); 2 Department of Pharmacognosy, Faculty of Pharmacy, Fayoum University, Fayoum 63514, Egypt; mhm07@fayoum.edu.eg; 3 Department of Pharmacognosy, Faculty of Pharmacy, Beni-Suef University, Beni-Suef 62514, Egypt; m.rateb11@aberdeen.ac.uk; 4 School of Computing, Engineering & Physical Sciences, University of the West of Scotland, Paisley PA1 2BE, UK; 5 Department of Pharmaceutical Chemistry, Faculty of Pharmacy, Nahda University, Beni-Suef 62511, Egypt; asmaa.aboulmaged@nub.edu.eg; 6 Department of Pharmacognosy, Faculty of Pharmacy, Mansoura University, Mansoura 35516, Egypt; faridbadria@gmail.com; 7 Department of Pharmacognosy, Faculty of Pharmacy, Minia University, Minia 61519, Egypt; usama.ramadan@mu.edu.eg; 8 Department of Pharmacognosy, Faculty of Pharmacy, Deraya University, 7 Universities Zone, New Minia City 1111, Egypt; 9 Department of Pharmacognosy, College of Pharmacy, King Khalid University, Abha 61441, Saudi Arabia; 10 Faculty of Applied Medical Sciences, Department of Medical Laboratory Technology, King Abdulaziz University, P.O. Box 80402, Jeddah 21589, Saudi Arabia; 11 Special Infectious Agents Unit, King Fahd Medical Research Centre, King Abdulaziz University, P.O. Box 80402, Jeddah 21589, Saudi Arabia

**Keywords:** hyaluronidase inhibitors, *Ravenala madagascariensis*, metabolomics, docking

## Abstract

Hyaluronidase enzyme (HAase) has a role in the dissolution or disintegration of hyaluronic acid (HA) and in maintaining the heathy state of skin. Bioassay-guided fractionation of *Ravenala madagascariensis* (Sonn.) organ extracts (leaf, flower, stem, and root) testing for hyaluronidase inhibition was performed followed by metabolic profiling using LC–HRMS. Additionally, a hyaluronidase docking study was achieved using Molecular Operating Environment (MOE). Results showed that the crude hydroalcoholic (70% EtOH) extract of the leaves as well as its *n*-butanol (*n*-BuOH) partition showed higher HAase activity with 64.3% inhibition. Metabolic analysis of *R. madagascariensis* resulted in the identification of 19 phenolic compounds ranging from different chemical classes (flavone glycosides, flavonol glycosides, and flavanol aglycones). Bioassay-guided purification of the leaf *n*-BuOH partition led to the isolation of seven compounds that were identified as narcissin, rutin, epiafzelechin, epicatechin, isorhamnetin 7-*O*-glucoside, kaempferol, and isorhamnetin-7-*O*-rutinoside. The docking study showed that narcissin, rutin, and quercetin 3-*O*-glucoside all interact with HAase through hydrogen bonding with the Asp111, Gln271, and/or Glu113 residues. Our results highlight *Ravenala madagascariensis* and its flavonoids as promising hyaluronidase inhibitors in natural cosmetology preparations for skin care.

## 1. Introduction

The skin is the external or exterior organ of our bodies which plays as a fence against chemical, physical, and biological pollutants [1]. Skin aging is the combination of natural aging with photo aging, where both include physical changes, atrophy of extracellular matrix in the dermal and epidermal layers which causes visible signs on the skin’s surface [2,3]. The dermal parts of the skin are responsible for keeping its firmness, flexibility, and moisture retention and are constantly being broke down by enzymes like collagenase, elastase, and hyaluronidase, leading to wrinkle forming, furthermore, free radicals and other oxidants speed this process. Thus, components that suppress the enzymes or play as antioxidants reduce wrinkle forming [4]. Hyaluronidase enzyme is a mucopolysaccharide-hydrolyzing enzyme that causes the degradation of hyaluronic acid (HA), an important small molecule, which fills the space between collagen and elastin fibers, and helps maintain skin moisture levels [5,6,7,8,9]. It serves as an integral part of the extracellular matrix and is responsible for improving hydration which improves the appearance of fine lines and wrinkles [6,10,11,12]. Inhibition of hyaluronidase enzyme (HAase) is potent at regulating agents, which are important for maintaining the balance between catabolism and anabolism of HA. There are many chemical classes such as alkaloids, polyphenols, flavonoids, and terpenoids that have shown HAase inhibition [13,14,15,16]. Earlier studies proved that the efficient HAase’s inhibitory ability of some flavonoids, like apigenin, luteolin, quercetin, kaempferol, and rutin is due to their closely related structure [17], on the other hand the activity towards Hyal increases with the increasing the number of hydroxyl groups, especially in the 2,3′ position (quercetin) and 5′(myricetin) and decreased after glycosylation or substitution of hydroxyl groups [13,14]. Also, natural extracts such as brown seaweed (*Padina pavonica*) have shown an inhibitory effect on hyaluronidase and is used as an anti-aging product [18]. The methanolic extracts of *Malaxis acuminate* leaves and stems exhibited photo protective activity against UV-A and UV-B radiation in addition to acting as HAase inhibitors. GC–MS of both leaf and stem extracts showed the presence of phenolic acids, sterols, α-hydroxy acids, amino acids, sugars, and glycosides which coincides with the use of *M. acuminata* as a rejuvenating remedy in several Ayurvedic formulations [19]. The *n*-butanol, aqueous and methanol extracts of *Pleurotus citrinopileatus* were also found to act as hyaluronidase inhibitors by 9.7%, 10.8%, and 25.4%, at concentrations of 2.0, 1.1, and 4.1 mg/mL, respectively, the aqueous and methanolic extract of *Pleurotus tuber-regium* showed hyaluronidase inhibition by 22.19% and 3.94%, respectively, at 100 µg/mL while *Trametes lactinea* aqueous and acetonic extract inhibited hyaluronidase by 88.6% and 88.3%, respectively [6]. The number of free phenolic and ortho-dihydroxyphenolic groups in molecules increased their effects in both DPPH and HAase assays [20]. Positive correlations between hyaluronidase inhibitory activity, total phenolic content, and total flavonoids were linked to the presence of polyphenolics and flavonoids [14,21].

On the other hand, abundant varieties of phytochemicals such as epicatechin, ziyuglycoside I, asiaticoside, aloin, ginsenoside, gallic acid, hydroxychavicol, hydroxybenzoic acid, hydroxycinnamic acid, magnolol, and curcumin act as free radical scavengers which prevent transdermal loss of water to keep skin free from wrinkles, ultimately leading to younger and healthier skin [22,23].

*Ravenala madagascariensis*, commonly called traveler’s tree or traveler’s palm, belongs to the family Strelitziaceae [24]. It is indigenous to Madagascar and is easily cultivated in the tropical regions, in particular as an ornamental tree [25]. The major classes of the chemical constituents identified so far in *R. madagascariensis* are flavonoids, tannins, saponins, terpenoids, and steroids [26]. Its hydro-alcoholic extract is used as a hydrating agent in cosmetic products, to achieve proper skin hydration [27]. In this study, varying plant organ extracts (leaf, flower, stem, and root) of *R. madagascariensis* were investigated for their HAase inhibitory activity. Subsequently, bioassay-guided fractionation and isolation of the major bioactive compounds was carried out leading to the rediscovery of multiple known compounds. Moreover, the interaction of isolated compounds to the HAase via docking studies was carried out.

## 2. Results

The crude extracts of different organs (leaf, flower, stem, and root) were subjected to hyaluronidase inhibition activity (Table 1), where the crude extract of the leaves was the most active HAase inhibitor (78.9%). These findings of the crude extracts of leaves and stems were much better that previously reported when compared with the aqueous and methanolic extract of *Pleurotus tuber-regium* that showed inhibiton by 22.19% and 3.94%, respectively at 100 µg/mL while it showed comparable activities with those of *Trametes lactinea* aqueous and acetonic extract that inhibited hyaluronidase by 88.6% and 88.3%, respectively [6]. Additionally, partitions of the leaves were evaluated for their HAase inhibition activity, where hydro-alcoholic as well as its *n*-BuOH extracts of *R. madagascariensis* showed higher inhibition (90% and 64.3%, respectively). The *n*-BuOH was subjected to chromatographic purification, yielding seven compounds identified as narcissin, rutin, epicatechin, isorhamnetin 7-O-glucoside, isorhamnetin 7-*O*-rutinoside, epiafzelechin, and kaempferol. The isolated compounds were tested for their inhibition percentage (Table 1). Results showed that epiafzelechin and epicatechin the highest inhibition value with 36.5% and 34.4%, respectively. While the other isolated ones are less, these results were going with previously reported that increasing the number of hydroxyl groups, especially in 2,3′ and 5′(myricetin) and decreased after glycosylation or substitution of hydroxyl groups. [13,14]. The Docking studies were carried out to describe the interaction properties between the isolated ones and the enzyme, showed that narcissin, rutin, and Quercetin 3-*O*-glucoside interact with HAase through the formation of hydrogen bonds with the Asp111, Gln271, and/or Glu113 residues.

### 2.1. Metabolic Profiling

**Figure 1:**
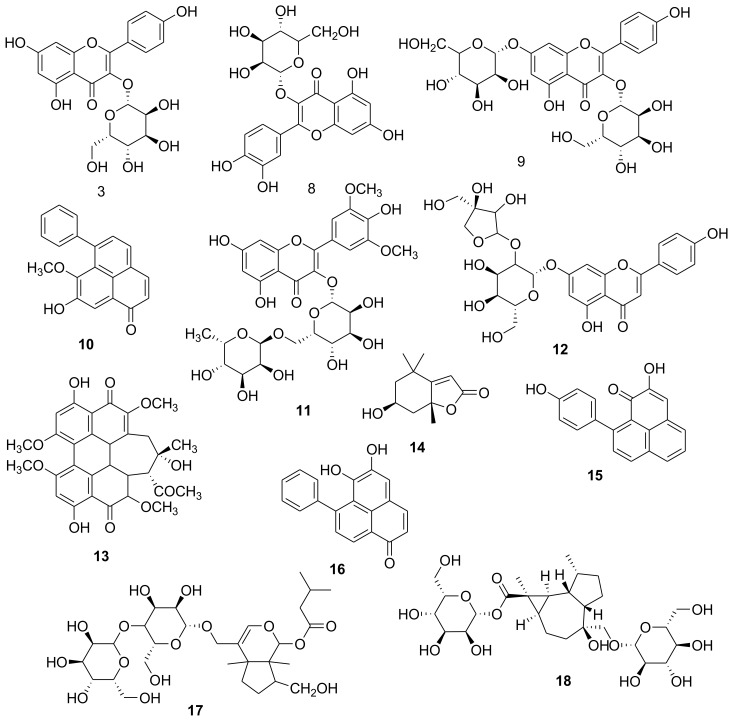
Structures of the dereplicated metabolites from *Ravenala madagascariensis.*

Chemical profiling of the secondary metabolites present in *R. madagascariensis* leaves was carried out using LC-HRESIMS for dereplication purposes. The results showed a diversity of constituents ranging from different chemical classes, where flavonoids predominated. The identified compounds (Figure 1) were tentatively identified by multiple databases (LipidMaps, METLIN and DNP databases). From these, the peak with *m/z* 624.391, with a molecular formula C28H32O16, was dereplicated as the flavonoid, glycoside isorhamnetin-3-*O*-rutinoside (narcissin) (**1**), which was previously isolated from *R. madagascariensis* [28], while the peak with *m/z* 611.163, with a molecular formula C_27_H_30_O_16_, was identified as quercetin-3-*O*-rutinoside (rutin) (**2**), previously isolated from *R. madagascariensis* [28]. The peak with *m/z* 816.209, with a proposed molecular formula C_38_H_40_O_20_, was specified as kaempferol-3-*O*-glycoside (**3**), already informed from close species of Zingiberales [29]. The peak at *m/z* 832.206, with an expected molecular formula C_38_H_40_O_21_, was dereplicated as quercetin-3-*O*-glucoside (**4**), which was previously identified from *R. madagascariensis* [28]. The peak at *m/z* 1090.314, with the suggested molecular formula C_50_H_58_O_27_, was dereplicated as kaempferol-3,7-diglycoside (**5**). The peak at *m/z* 302.101, with a suggested as molecular formula C_20_H_14_O_3_, was dereplicated as 8-hydroxy-7-methoxy-6-phenylphenalen-1-one (**6**) which was previously isolated from genus of Strelitzia, family Strelitziaceae [30]. Similarly, a flavone glycoside with the molecular formula C_35_H_44_O_22_, was distinguished as 3,4′,5,7-Tetrahydroxy-3′,5′-dimethoxyflavone-3-*O*-α-l-rhamnopyranoside (**7**) from the peak at *m/z* 816.232 [31]. The experimental results revealed that kaempferol, quercetin, rutin, apiin, and isorhamnetin interact with hyaluronidase, induce conformational changes and therefore inhibit hyaluronidase catalytic activity (M. Liu et al., 2013). Additionally, related compounds identified for the first time from this species were also characterized as apiin (**8**), shiraiachrome A (**9**), loliolide (**10**), hydroxyanigorufone (**11**), lachnanthocarpone (**12**), confertoside (**13**), dendroside D (**14**) from the family, based on their *m/z* 564.157, 546.161, 196.118, 288.087, 288.087, 608.273, 592.278, respectively and in accordance with the molecular formulas C_26_H_28_O_14_, C_30_H_26_O_10_, C_11_H_16_O_3_, C_19_H_12_O_3_, C_19_H_12_O_3_, C_27_H_44_O_15_, C_27_H_44_O_14_, respectively.

### 2.2. Identification of Purified Compounds

**Figure 2:**
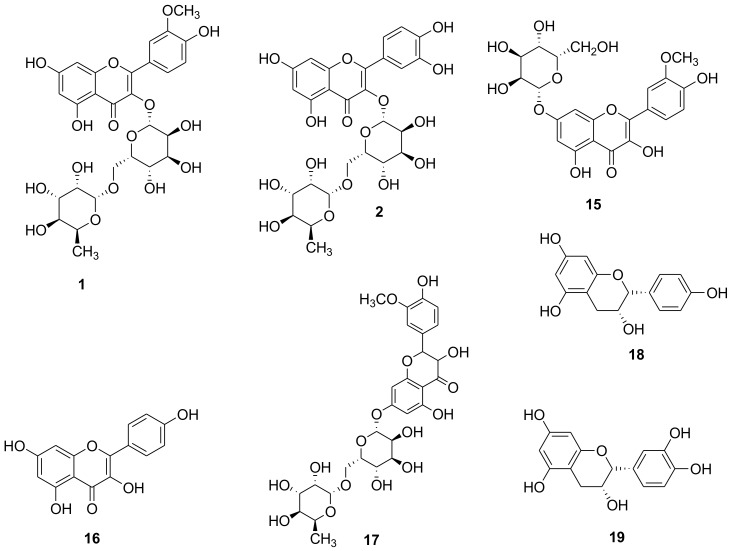
The isolated compounds structures from *Ravenala madagascariensis*.

The isolated compounds (Figure 2) were structurally elucidated established on their physiochemical, chromatographic properties, and spectroscopic analysis (UV, ESI-MS, ^1^H-NMR and ^13^C-NMR), as well as comparison to standards. The isolated compound **1** was obtained as yellow amorphous powder, dark purple color under UV light which changed to bright yellow with ammonia vapor. R_f_ = 0.38 by using CH_2_Cl_2_/ MeOH (8:2) as solvent system for TLC. ^1^H-NMR: (400 MHz, MeOD), δ 7.84 (1H, s, H-2′), δ 7.52 (1H, d, *J* = 8.0 Hz, H-6′), δ 6.95 (1H, d, *J* = 8.0 Hz, H-5′), δ 6.4 (1H, s, H-8), δ 6.2 (1H, s, H-6), δ 5.2 (1H, d, *J* = 7.5 Hz, H-1′′), δ 4.5 (1H, bs, H-1′″), δ 3.83 (3H, s, OMe-3′), δ3.04–3.85 (8H, m, H-2′′, H-3′′, H-4′′, H-5′′, H-2′″, H-3′″, H-4′″, H-5′″), δ 1.1 (3H, d, *J* = 6 Hz, CH-6′″). ^13^C-NMR: (100 MHz, MeOD), 177.9, 164.6, 157.5, 157.1, 149.4, 146.9, 134.0, 122.6, 121.6, 114.7, 113.1, 104.3, 103.0, 102.0, 98.6, 93.5, 76.7, 75.9, 74.5, 72.4, 70.9, 70.7, 70.2, 68.4, 67.1, 55.6, 16.6. From previous data it was identified as narcissin [28,32].

Compound **2** was the same as **1** except the absence of OCH_3_ signals at δ 3.83 and 55.6 and was identified as rutin [31], compound **15** was obtained as yellow amorphous powder, dark purple color under UV light which changed to bright yellow with ammonia vapor. R_f_ = 0.18 by using CHCl_3_/ MeOH (9.5:0.5) as solvent system for TLC. ^1^H-NMR: (400 MHz, MeOD), δ 7.93 (1H, s, H-2′), δ 7.79 (1H, d, *J* = 8.0 Hz, H-6′), δ 6.94 (1H, d, *J* = 8.0 Hz, H-5′), δ 6.83 (1H, s, H-8), δ 6.49 (1H, s, H-6), δ 5.05 (1H, d, *J* = 7.2 Hz, H-1′′), δ 3.96 (3H, s, OMe-3′), δ 3.04–3.85 (5H, m, H-2′′, H-3′′, H-4′′, H-5′′, H-6′′). ^13^C-NMR: (100 MHz, MeOD), 177.9, 163.3, 161.2, 158.1, 156.6, 149.5, 147.3, 131.6, 122.6, 120.7, 114.8, 113.2, 106.0, 100.3, 101.1, 98.8, 92.6, 77.0, 76.5, 73.3, 69.7, 62.6, 55.1, as isorhamnetin-7-*O*-glucoside which [33,34], while compound **16** was obtained as yellow amorphous powder, dark purple color under UV light which changed to bright yellow with ammonia vapor. R_f_ = 0.14 by using CHCl_3_/ MeOH (9.8:0.2) as solvent system for TLC. ^1^H-NMR: (400 MHz, MeOD), δ 8.09 (1H, d, *J* = 8.0 Hz, H-2′, H-6′), δ 6.94 (1H, d, *J* = 8.0 Hz, H-3′,H-5′), δ 6.43 (1H, s, H-8), δ 6.21(1H, s, H-6), ^13^C-NMR: (100 MHz, MeOD), 177.9, 165.4, 161.5, 158.2, 157.4, 131.3, 129.3, 122.3, 115.0, 104.5, 98.9, 93.3 as kaempferol [35,36].

Compound **17** was obtained as yellow amorphous powder, dark purple color under UV light which changed to bright yellow with ammonia vapor. R_f_ = 0.16 using CH_2_Cl_2_/ MeOH (7.5:2.5) as solvent system for TLC. ^1^H-NMR: (400 MHz, MeOD), δ 7.96 (1H, s, H-2′), δ 7.67 (1H, d, *J* = 8.0 Hz, H-6′), δ 6.95 (1H, d, *J* = 8.0 Hz, H-5′), δ 6.8 (1H, s, H-8), δ 6.51 (1H, s, H-6), δ 5.2 (1H, d, *J* = 7.5 Hz, H-1′′), δ 5.09 (1H, bs, H-1′″), δ 3.83 (3H, s, OMe-3′), δ 3.04–3.85 (8H, m, H-2′′, H-3′′, H-4′′, H-5′′, H-2′″, H-3′″, H-4′″, H-5′″), δ 1.1 (3H, d, *J* = 6 Hz, CH-6′″). ^13^C-NMR: (100 MHz, MeOD), 177.9, 163.3, 161.2, 158.1, 156.6, 149.6, 146.9, 134.19, 122.9, 121.4, 114.7, 113.2, 105.8, 102.6, 101.1, 99.6, 94.6, 76.7, 75.9, 74.5, 72.4, 70.9, 70.7, 70.2, 68.4, 67.3, 55.4, 16.5 as isorhamnetin-7-*O*-rutinoside [34], on the other hand compound **18**, was obtained as a colorless needle crystals, dark purple color under UV light gave red with Vanillin/ H_2_SO_4_, R_f_ = 0.5 by using CH_2_Cl_2_/ MeOH (8.5:1.5) as solvent system for TLC. ^1^H-NMR: (400 MHz, MeOD), δ 7.3 (1H, d, *J* = 8.5 Hz, H-2′, H-6′), δ 6.8 (1H, d, *J* = 8.6 Hz, H-5′, H-3′), δ 5.99 (1H, d, *J* = 2.2 Hz, H-8),δ 5.94 (1H, d, *J* = 1.5 Hz, H-6), δ 4.8 (1H, s, H-2), δ 4.2 (1H, m, H-3), δ2.9, 2.88 (1H, dd, *J* = 16.8, 4.5 Hz, H-4a, H-4b). ^13^C-NMR: (100 MHz, MeOD), 27.9, 66.1, 78.5, 94.6, 95.1, 98.8, 114.4, 127.8, 127.9, 130.2, 156.0, 156.2, 156.4, as epiafzelechin [37], and compound **19** was the same with only one missing aromatic proton at position 3` at δ 6.8 and was identified as epicatechin [38,39].

### 2.3. Modeling Study Exhibiting the Binding Ability of Polyphenolic Compounds to HAase

**Figure 3:**
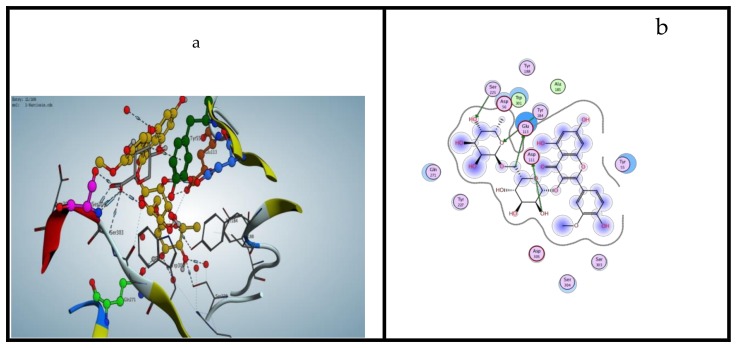

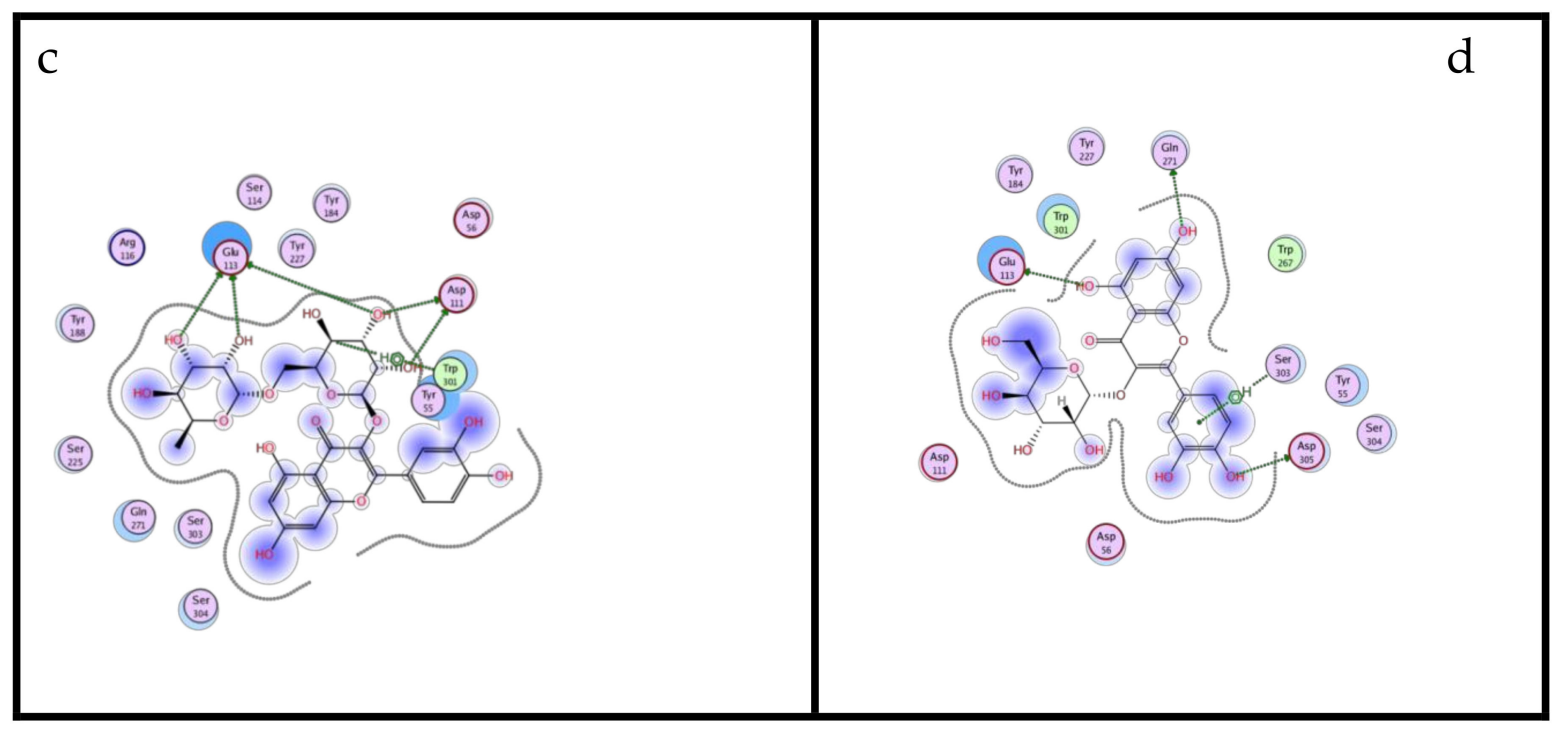
Docked poses relative or proportional to the minimum energy configuration for narcissin, rutin & quercetin binding to HAase. (Panel **a**) Binding pattern of narcissin colored by element, ball and stick into HAase showing 4 hydrogen bond interactions (dotted lines). (Panel **b**–**d**) displayed the most active flavonoids narcissin (**b**), rutin (**c**) and quercetin (**d**).

To predict the precise binding sites on HAase and explore the interaction between the target compounds and HAase systematically, a docking study was run using Molecular Operating Environment (MOE; Chemical Computing Group Inc., Montreal, Canada). From the docking calculation, the smallest energy-ranked results of the target compounds -HAase conformations are outlined in Table 2. By comparing, of the data from Table 1 with the data from Table 2, the noticed free energy change (ΔG°) for the target compounds -HAase systems was consistent with the results of biological studies. As shown in Figure 3, all the compounds containing the flavonoid nucleus were situated in the hydrophobic cavity of HAase and were encircled by hydrophilic and hydrophobic amino acids. Therefore, mainly the interaction between flavonoids and HAase is due to electrostatic forces and hydrophobic interactions. However, due to more hydrophobic amino acids lining quercetin in the binding site, hydrophilic interaction was more conspicuous than electrostatic force in the quercetin -HAase system. As shown in Figure 3, many amino acid residues, such as Ser303, Gln271, Glu113, Tyr227, Asp111, Tyr55 and Ser304, manifested in the binding of each compound with HAase [40]. These results indicate that these residues would perform a significant role in the interaction between the illustrated compounds and HAase and might constitute the catalytic site of HAase. Furthermore, due to the presence of different ionic and polar groups, there are also large numbers of hydrogen bonds in HAase binding site. As shown in the following figures, one of the hydrogen bonds is formed with the Asp111, Gln271 and/or Glu113 residues in the flavonoid (narcissin, rutin and quercetin-3-O-glucoside) -HAase systems (Figure 3). The compounds that exhibited the lowest binding free energy values were rutin and narcissin with values −7.119 Kcal/mol and −6.853 Kcal/mol, respectively, which agrees with their inhibitory effect on HAase. Therefore, according to docking studies and the biological screening, it could be suggested that flavonoid or flavonoid-like compounds have the ability bind to the HAase catalytic site, which would suppress the activity of the enzyme.

Structure activity relationship showed that: The C2,3 double bonded flavonoids have high potency [13,14], the flavonoid glycosides were more potent in their inhibitory effects than aglycone and the ortho-dihydroxyl substitution exhibited greater inhibition than those singly hydroxylated, while the methoxy substitution decreased the inhibitory effects. The following flavonoid structure conferred potent inhibitory effect: the C2,3 double bond and ortho-dihydroxyl substitution.

## 3. Materials and Methods

### 3.1. Plant Material

*Ravenala madagascariensis* (Sonn.) leaves, flowers, stem and root were collected from the El Orman garden, Egypt, in August 2015. The plant was authenticated by Professor Ibrahim Ahmed El-Garf (Department of Botany and Microbiology, Faculty of Science, Cairo University, Giza, Egypt). The leaves were air-dried, powdered, and kept in amber-colored, air-tight glass containers at low temperature for preservation of chemical materials for future testing.

### 3.2. Chemicals and Reagents

Brain heart infusion media, agar, sodium hyaluronidate, human albumin, sheep blood agar, incubator, *Staphylococcus aureus*, glacial acetic acid, Tryptic soya broth (TSB), incubator, autoclave, filters (pore size, 0.22 μm), 70% ethanol (EtOH), distilled H2O, Hyaluronic acid, 5% DMSO, methanol, sodium tetraborate, standard inhibitor (Tannic acid and p-dimethyl amino benzaldehyde (PDMAB). All chemical substances and standards were provided from Sigma-Aldrich (St Louis, MO, USA).

### 3.3. Extraction and Isolation

Dried plant material was detached into leaves, flowers, stem and root and then ground. Subsequently, each part was extracted with 70% ethanol (EtOH) by cold maceration, and then concentrated under vacuum. The powdered leaves (1.7 kg) were extracted exhaustively at room temperature then concentrated to yield 400 g. Approximately half of this extract (~200 g) was resuspended in distilled H2O and successively fractionated with solvents of increasing polarity: n-hexanes, dichloromethane (DCM), ethyl acetate (EtOAc) and finally *n*-BuOH.

The *n*-BuOH extract (25 g) was undergone to a polyamide column (100 × 5 cm, 250 g) with an increasing elution gradient of MeOH: H2O in increments of 10% more H2O. Fractions (100 mL each) were collected, evaporated under reduced pressure and screened by TLC using various solvent systems. Similar fractions were pooled together yielding 12 fractions. The fourth fraction was subjected to a silica gel column (60 × 3 cm, 100 g) with a gradient eluting from DCM: MeOH yielding a further 21 subfractions. Compounds 1 (10 mg), 2 (10 mg) and 3 (20 mg) were isolated from the sixth, eighth and tenth subfractions, respectively, by further separation using a sephadex LH-20 column (38 × 3 cm, 40 g) with an isocratic elution of MeOH: H2O (80:20) The ninth fraction was submitted to a silica gel column (60 × 3 cm, 100 g) with a gradient eluting from DCM: MeOH followed by a sephadex LH-20 column (38 × 3 cm, 40 g) eluted with 80% MeOH to give (4, 20 mg) and (5, 25 mg). Finally, the tenth fraction was separated by a sephadex LH-20 column (38 × 3 cm, 40 g) with a 80% MeOH isocratic elution subsequently followed by another sephadex LH-20 column (38 × 3 cm, 40 g) isocratic elution with saturated n-butanol to give (6, 15 mg) and (7, 25 mg).

### 3.4. Biological Activity Study

#### 3.4.1. Preparation of Hyaluronidase

Following the procedure laid out by Smith et al., 1968 [41], 100 mL of brain heart infusion media was prepared with 1 g of agar, autoclaved for 15 min at 121 °C, and cooled to 46 °C; then, a 2 mg/mL solution of sodium hyaluronidate, was added to the cooled medium to a final concentration of 400 μg/mL. 5% human albumin was added under constant mixing to give a final concentration of 1%. Plates were then poured to a depth of 3–4 mm and left to solidify at 4 °C. The isolates of *Staphylococcus aureus* were grown on sheep blood agar until growth was clearly observed. A single colony was streaked onto the hyaluronic acid medium, incubated at 37 °C, and observed daily until growth was detected (24 to 72 h). On the day growth was first observed, the plate was immersed with 2N glacial acetic acid which binds hyaluronic acid and albumin, forming a white precipitate. Hyaluronidase production was confirmed if a clear zone was observed.

#### 3.4.2. Hyaluronidase Inhibition Assay

The strains of *Staphylococcus aureus* were expanded overnight in TSB at 37 °C with shaking, then sub-cultured 1:1000 and grown at 37 °C for the time specified below. Spent culture medium was isolated with Spin-X filters (pore size, 0.22 μm) then frozen at −20 °C until used in biological assays. 50 μL of spent culture medium was incubated for 15 min at 37 °C with 50 μL of the used test inhibitor (1 mg/mL dissolved in 5% DMSO). Hyaluronic acid (HA) (100 μL at 1 mg/mL) was blended and permitted to react at 37 °C for 15 min. To stop the reaction 25 μL of sodium tetraborate solution (0.8 M, pH 9.1) was added and then the reaction mixture was vortexed and boiled for 3 min. The positive control (tannic acid, was used as HAase inhibitor) [42,43,44] and negative control were made using the identical procedure but without the test or standard inhibitor (50 μL of 5% DMSO). In parallel, 50 μL of 5% DMSO and 50 μL spent medium were added to 125 μL of stop solution (1 mg/mL hyaluronic acid, 0.8 M potassium tetraborate, pH 9.1) at time zero, vortexed, and boiled for 3 min. The samples were distributed into a 96-well microtiter plate in quadruplicate. Freshly prepared PDMAB solution (10% [*w*/*v*] p-dimethyl amino benzaldehyde, 12.5% [*v*/*v*] 10M HCl, and 87.5% [*v*/*v*] glacial acetic acid) was added to each well. The plate was incubated at 37 °C for 20 min to let the color change. The absorbance at 590 nm was evaluated by using a microplate reader

% inhibition = (A_o_ − A1)/A_o_ × 100 (1)

where A1 is the absorbance of the standard/ extracts, A_o_ is the absorbance of the control.

### 3.5. Metabolic Profiling

The crude extracts of *R. madagascariensis* (leaves, flowers, stem, and root) were undergone to metabolomic using LC-HRESIMS analytical methods as stated by Abdel Mohsen et al. [45]. Each extract (1 mg/mL dissolved in MeOH) was analyzed separately on an Accela HPLC with an Accela UV-Vis coupled to an Exactive (Orbitrap) MS spectrometer from Thermo Fisher Scientific (Berman, Germany). Purified water (a) and acetonitrile (b) used as mobile phase with 0.1% formic acid in both. The gradient began at 10% (b) to 100% (b) in 30 min and kept isocratic elution for the next 5 min before back to 10% for 1 min (flow rate of 300 µL/ min). Then mobile phase was re-equilibrated for 9 min previous to the following injection. By using untargeted Higher-energy Collision Dissociation (HCD) mechanism, the range of mass was set from *m/z* 50–1000 for MS/MS and *m*/*z* 100–2000 for ESI-MS using in source collision-induced dissociation (CID). The raw data was derived in MZmine 2.12, a framework for differential analysis of MS data. The individual peaks were detected by chromatogram deconvolution. The retention time normalizer was applied for chromatographic alignment and gap-filling. An Excel macro was applied on the positive ionization mode data files produced by MZmine to extract the peaks from each samples and dereplicated each ion peak with compounds in the customized database (using RT and *m*/*z* threshold of ±5 ppm), which supplied details on the assumed identities of metabolites in all extracts. By comparison with LipidMaps, DNP and METLIN databases, 19 metabolites were identified.

### 3.6. Molecular Docking Investigation

Docking calculations were achieved using (MOE) Molecular Operating Environment on a HAase model (PDB code 1FCV, http://www.rcsb.org/pdb/home/home.do). The structures of the compounds were generated by Chemdraw Ultra 8.0. Docking emulation was achieved on the identified compounds with the next protocol: (1) Enzyme structures were examined for missing atoms, bonds and contacts, as well as (2) hydrogen atoms were joined to the enzyme structure. (3) Then by using the builder module, the ligand molecules were established and were energy minimized. (4) The active site was created using the MOE Alpha Site Finder. (5) Ligands were docked within the HAase active site using MOE Dock with simulated annealing utilized as the search protocol and CHARMm molecular mechanics force field. (6) The smallest energy conformation of the docked ligand complex was chosen and undergone to more energy minimization using CHARMm force field. Determination of precision of this docking protocol was carried out by redocking the co-crystallized ligand into the HAase vital site. This method was rerun triple times and the best ranked solutions of the ligand displayed RMSD values of 1.84 A° from the site of the co-crystallized ligand for HAase. Generally, RMSD values smaller than 2.0 A° show that the docking protocol is efficient to accurately promising the binding orientation of the co-crystallized ligand [46]. This protocol was deemed to be convenient for the docking of the test compounds into the active site model of HAase.

## 4. Conclusions

This work showed the importance of natural products specially phenols and polyphenols as a competitive inhibitor of hyaluronidase as a key enzyme in healthiness of skin. These findings may assist for the application of plant under investigation in future in cosmetic and in phototherapy as a dietary supplement with fewer side effects. On the other hand, it gave insight about the proposed interactive sites between compounds and the enzyme that may help in optimization of these compounds for production of more active compounds.

## Figures and Tables

**Table 1:** Hyaluronidase inhibition of *Ravenala madagascariensis* ethanolic extracts and pure compounds isolated in this study.

Crude Extracts	Hyaluronidase Inhibition	Leaf Partitions	Hyaluronidase Inhibition	Pure Compounds	Hyaluronidase Inhibition
**Leaves**	78.9% ± 0.023	***n-*Hexane**	28.6% ± 0.065	**Narcissin**	27.2% ± 0.043
**Stem**	56.1% ± 1.082	**DCM**	3.8% ± 1.044	**Rutin**	23.7% ± 01.070
**Flower**	9.4% ± 0.1012	**EtOAc**	32% ± 0.1334	**Epicatechin**	34.4% ± 0.038
**Root**	20.5% ± 0.006	***n-*BuOH**	64.3% ± 0.015	**Epiafzelechin**	36.5% ± 0.045
**Tannic acid**	95.4% ± 0.006	**total 70% EtOH**	90 % ± 0.006	**Isorhamnetin 7-*O*-glucoside**	11.4% ± 0.022
				**Kaempferol**	8.7% ± 0.039

**Table 2:** The lowest energy ranked results of target compounds HAase binding configurations.

Compound	Score	Average Number of Poses Per Run
**Narcissin**	−6.853	10
**Quercetin-3-O-glucoside**	−6.088	10
**Rutin**	−7.119	10
**8-hydroxy-7-methoxy-6-phenylphenalen-1-one**	−4.54	10
**Hydroxyanigorufone**	−4.306	7
**Epiafzelechin**	−4.436	8
**Epicatechin**	−4.852	8
**Lachnanthocarpone**	−4.539	6
**Confertoside**	−5.936	10
**Dendroside D**	−6.701	2
**Loliolide**	−4.021	8
**Shiraiachrome A**	−6.08	9

The displayed score is the mean of 3 sequential runs. The docking technique was validated by successful pose-retrieval docking trial of the ligand (score: −5.370).
